# Predictors of suicide attempters in substance-dependent patients: a six-year prospective follow-up

**DOI:** 10.1186/1745-0179-3-20

**Published:** 2007-10-10

**Authors:** Kjell Bakken, Per Vaglum

**Affiliations:** 1 Centre for Addiction Issues, Department for Substance Abuse, Innlandet Hospital Trust, Norway; 2 Department of Behavioural Sciences in Medicine, Faculty of Medicine, University of Oslo, Norway

## Abstract

**Background:**

This is a six-year prospective follow-up of a former cross sectional study of suicide attempters in a sample of treatment-seeking substance-dependent patients. The aims were to explore the frequency of patients with new suicide attempts (SA) during the six-year observation period, and to explore the predictive value of lifetime Axis I and II disorders, measured at index admission, on SA in the observation period, when age, gender and substance-use variables, measured both at admission and at follow-up, were controlled for.

**Methods:**

A consecutive sample of 156 alcohol-dependent and 131 poly-substance-dependent inpatients and outpatients in two Norwegian counties were assessed at index admission (T1) with the Composite International Diagnostic Interview (Axis I disorders), Mon's Clinical Multiaxial Inventory (Axis II disorders) and Hopkins Symptom Checklist-25 (mental distress). At follow-up six years later (T2), 56% (160/287 subjects, 29% women) were assessed using the HSCL-25 and measures of harmful substance use (Alcohol Use Disorders Identification Test and Drug Use Disorders Identification Test).

**Results:**

The prevalence of patients with SA between T1 and T2 was 19% (30/160), with no difference between sexes or between patient type (alcohol-dependent versus poly-substance-dependent). Sober patients also attempted suicide. At the index admission, lifetime eating disorders, agoraphobia with and without panic disorder, and major depression were significantly and independently associated with SA. Prospectively, only lifetime dysthymia increased the risk of SA during the following six years, whereas lifetime generalized anxiety disorder reduced the risk of SA. Individually, neither the numbers of Axis I and Axis II disorders nor the sum of these disorders were independently related to SA in the observation period. Substance use measured at T1 did not predict SA in the follow-up period, nor did harmful use of substances at follow-up or in the preceding year.

**Conclusion:**

A high prevalence of SA was found six years later, both in patients still abusing substances and in sober patients. To prevent SA, treatment of both affective disorders and substance abuse is important.

## Background

Clinical studies have shown lifetime prevalence of suicide attempts (SA) among substance use disorders (SUD) patients to vary from 16% to 71% [1-3], compared with 3–5% in the general population [4,5]. Elevated SA rates have been seen in patients with earlier onset of SUD, long-lasting substance misuse [5-11], heavier drinkers [7,8,12,13] and those who abuse a higher number of substances [3,5,14-19].

In SUD patients, major depression seems to be strongly associated with SA [4,6,11-17,20-25]. Anxiety disorders are also associated with SA in SUD patients [6,7,23,25,26], although there are contradicting results regarding the association between SA and anxiety disorders in general population-based studies [27-29]. In some cross sectional clinical studies, an increasing number of comorbid disorders [7,16,26,30], and in particular, the combination of major depression and anxiety disorder, increased the probability of suicide attempts [23], as in general population-based studies [27,28].

Comorbidity of two or more Axis I disorders, or Axis I and Axis II disorders, has been shown to be associated with suicide attempts in both cross sectional studies [4,7,26,31,32] and in prospective studies [3,6,33]. Driessen and colleagues [23] found the combination of Axis I and II disorders to be of particular importance in the retrospective part of their study, but in the prospective part, SA was not predicted by lifetime Axis I or Axis II disorders. Other prospective studies have found little association between SA and baseline Axis I disorders [17,34], and Axis II disorders [12]. This discrepancy may be due to the short observation time in the prospective studies, compared with the lifetime perspective in the retrospective studies.

Previous suicide attempts have consistently been reported to be a significant predictor of new suicide attempts in clinical samples with alcohol-dependent [17,23,35] and drug-dependent [12,18,19] patients. However, the impact of Axis I and II disorders on forthcoming SA and repeated SA in SUD patients is still unclear [23]. There is also no consistent answer to the very important question of whether recovery from SUD leads to a significant reduction in SA in prospective studies [17,20].

We have previously published a cross sectional study of a consecutive admission sample of SA patients, including men and women, and both alcoholics and drug addicts, in two counties in Norway. Nearly half (47%) of this sample reported lifetime SA [25], and this occurred more often in poly-substance-dependent patients than alcohol-dependent patients (58% vs 38%, respectively, *p *= 0.002). Early onset of SUD (<18 years), and long duration of SUD (>15 years), were found to be independent predictors of SA, together with lifetime eating disorder, major depression and agoraphobia (with and without panic disorder). It is of clinical importance to explore whether these predictors could be replicated in a long-term (six years) prospective study of SA in the same sample. There is a lack of long-term (>5 years) prospective studies of SA in treatment-seeking SUD patients that cover genders, alcoholics and illegal-drug addicts in the same study, using structured diagnostic interviews [36].

Based on this, we conducted a six-year prospective study of alcohol-dependent or poly-substance-dependent patients from nine inpatient and outpatient facilities in two counties in Norway. Information about socio-demographic, substance use, Axis I and II disorders and mental distress was gathered at the index admission through structured diagnostic interviews and well established self-report instruments. At follow-up six years later, a postal questionnaire was used to collect information about mental distress, suicide attempts and continuous substance abuse.

Our research questions are:

1. What is the prevalence of suicide attempters (SA) during the six-year observation period?

2. Do patients with no harmful use of substances at follow-up or in the preceding year attempt suicide less often than relapsers?

3. Which index admission factors (socio-demographic, lifetime Axis I and II disorders and substance use variables) are associated with attempting suicide during the six-year observation period?

4. Do the lifetime Axis I and II disorders measured at index admission have an independent impact on suicide attempts during the observation period when age, sex and SUD variables, measured at admission and at follow-up, are controlled for?

## Methods

### Sampling

A consecutive sample (n = 287) (30% women, mean age = 38.6 ± 11.3 years) of DSM-IV diagnosed substance-dependent patients (156 alcohol dependent and 131 poly-substance-dependent) from three outpatient (n = 157) and six inpatient (n = 130) public facilities in two Norwegian counties were recruited from the 690 patients who sought treatment between September 1997 and November 1998 (participation rate = 42% (287/690)). Inclusion criteria for patients from the outpatients' facilities were at least three consultations, whereas in the inpatients units, the patients had to stay at least for two weeks to be included in the study. Those who were not recruited either refused to participate, left treatment prematurely, or most often, did not receive a properly presented study proposal from the clinicians. Participants and non-participants did not differ significantly with regard to socio-demographic variables or substance-use variables, except that our sample was somewhat older than the non-participants (38.6 years vs 35.6 years, respectively, *p *< 0.001). Compared with a national sample (n = 5000) drawn from the entire Norwegian treatment-seeking population [37], our participants were somewhat older (23% vs 36% younger than 30 years, *p *< 0.001), more frequently abused alcohol (63% vs 50%, *p *< 0.001), and were more often married/cohabiting (36% vs 27%, *p *= 0.02). In general, our sample appeared somewhat skewed towards having fewer young drug addicts and more long-time substance abusers compared with the national sample. Sampling, participants and methods have been described more extensively in previous papers [38]. All patients gave their written informed consent to assessment at admission and contact at follow-up.

The National Registry provided data for deceased patients. Death certificates (cause of death) were obtained from the Cause of Death Registry (Division for Health Statistics in Statistics Norway). Eleven per cent (33/287) of patients had died, and information regarding causes of death was obtained for 26 of these. The deaths of 21 patients were substance-related. Of the remaining five patients, two died from cancer, one from suicide, and two for unknown reasons. A comparison of deceased patients and survivors is presented by Bakken et al. [39]. Of deceased patients, 36% had reported at least one SA at index admission, compared with 48% of survivors (*p *= 0.217).

The six-year follow-up questionnaire was mailed to the surviving individuals of the index admission sample (n = 254). Most of the patients were also contacted by telephone. Participants were paid 300 Norwegian kroner (approximately 38 Euros) for completing the form. Of the surviving patients (n = 254), 63% (n = 160, 29% women, mean age = 45.1 ± 11.2 years) returned the questionnaire and this constituted the follow-up sample. Of those who did not respond at follow-up (n = 94), 14 actively refused to participate, 23 were not located, and 57 received the assessment form twice (limit set by the Norwegian Data Inspectorate), but did not reply. The participation rate was similar for former patients from both outpatient and inpatient facilities. Our follow-up sample (n = 160) did not differ significantly from those who did not participate (n = 94) on any variable measured at admission.

### Evaluation at index admission (baseline, T1)

The rating instrument used to assess socio-demographic and treatment history was the Norwegian National Client Assessment form. Lifetime Axis I disorders were evaluated with a structured interview, the Composite International Diagnostic Interview (CIDI) [40]. Diagnosis of substance abuse, harmful use, and dependence was made with the CIDI (based on DSM-IV and ICD-10 criteria). ICD-10 diagnosis were used for the non-substance Axis I disorders. The CIDI has shown good feasibility in general populations and high inter-rater reliability, and has been subjected to tests of reliability and validity with satisfactory results [41]. We used it in a non-hierarchical way.

Lifetime suicide attempters were identified using one question from the Norwegian National Client Assessment Form "Has the client ever attempted suicide?" (n = 254) and one question from the module for major depressive episode in the CIDI interview (CIDI-M), "Have you ever attempted suicide?" (n = 33). Those who reported at least one lifetime suicide attempt in one of the two instruments were classified as SA. In total, 130 patients had attempted suicide before index admission, 150 had not, and there was a lack of information for the remaining seven patients.

Mental distress was measured with the self-report instrument, the Hopkins Symptom Checklist (HSCL-25) [42]. The HSCL-25 consists of 25 items that measure anxiety and depression symptoms that occurred during the previous week. The mean total sum score is called the General Symptom Index (GSI). Cronbach's alpha was 0.94.

Personality disorders were assessed using the self-report instrument, Millon's Clinical Multiaxial Inventory (MCMI-II) [43], a 175-item instrument, with response options of "true" and "false". This instrument measures 13 personality scales according to the DSM-III-R diagnostic system. Our cut-off value for "caseness" on the different MCMI-II scales was a base rate score of 85 or more. This is a more stringent diagnostic criterion than is recommended by the MCMI manual, but was used to ensure that the diagnostic criteria for any given personality disorder was satisfied. Findings from several studies of the psychometric properties of the MCMI-II have reported acceptable test-retest reliability, and generally acceptable levels of convergent and discriminant validity of the scales [44].

### Evaluation at six-year follow-up (T2) (n = 160)

Because of a lack of resources, it was not possible to conduct personal interviews (CIDI) to evaluate psychiatric diagnoses and SUD at follow-up. As an alternative, well-established self-report instruments were used. Mental distress at follow-up was measured with the same self-report instrument used at baseline, the HSCL-25. Cronbach's alpha was 0.95. Substance abuse was measured with two self-report instruments, the Alcohol Use Disorders Identification Test (AUDIT) and the Drug Use Disorders Identification Test (DUDIT), which covered substance use at follow-up and during the preceding year. These instruments have good reliability and validity [45,46]. AUDIT is a screening instrument for hazardous and harmful alcohol consumption [47]. It is a 10-item questionnaire that includes sections on alcohol consumption, alcohol dependence, and alcohol-related problems. Responses to each question are scored from 0 to 4, giving a maximum possible score of 40. Screening levels for hazardous alcohol use are ≥ 8 points for men and ≥ 6 points for women. In our study, reliability according to Cronbach's alpha was 0.94. DUDIT is an 11-item self-report instrument intended for use with AUDIT [46]. Screening levels for drug-related problems are ≥ 6 points for men and ≥ 2 for women from a maximum of 44 points. Cronbach's alpha was 0.95.

Patients were classified into one of two groups, "abstainers" or "relapsers", based on their AUDIT and DUDIT scores. The "abstainers" (n = 48) consisted of persons with no drug- or alcohol-related problems at follow-up or during the immediately preceding 12 months (based on an AUDIT score of <8 points for men and <6 points for women and a DUDIT score of <6 points for men and <2 points for women). Those who used legal methadone as the only substance at follow-up (n = 6), were also classified as "abstainers". The "relapsers" (n = 112) consisted of persons with drug- or alcohol-related problems during the preceding year and at follow-up (based on an AUDIT score of ≥ 8 for men and ≥ 6 for women, or a DUDIT score of ≥ 6 for men and ≥ 2 for women). The mean AUDIT score was 11.4 ± 10.5 and the mean DUDIT score was 5.8 ± 9.6.

Attempted suicide during the observation period was measured by the same question asked in the admission assessment, "Have you ever attempted suicide"? To identify attempts that occurred in the period between T1 and T2, another question was asked, "If you have attempted a suicide, approximately when did this happen. If there were multiple suicide attempts, please write down the year for each of them". In the follow-up sample (n = 160), 77 patients reported a lifetime suicide attempt when they were evaluated at index admission. An additional seven patients, who had said in the evaluation at index admission that they had not attempted any lifetime SA, stated in the follow-up evaluation that they had attempted suicide once before index admission. In total, this gave us 84 SA patients (77 + 7) at the index admission. The dependent variable in this study was the number of suicide attempters identified during the follow-up period (n = 30).

### Statistics

Differences in means between groups were assessed with analyses of variance (ANOVAs) and differences in categorical variables were measured with χ^2 ^tests and odds ratios. Bivariate correlations between continuous variables were measured using Pearson's product-moment correlation coefficient. Finally, multiple logistic regression analyses (method: enter, block-wise) were used to assess the controlled effects of predictors showing significant bivariate associations with SA in the observation period and controlling for harmful use of substances at follow-up or in the preceding year. All analyses were conducted using SPSS for Windows, version 14.0.

### Ethics

The study protocol was reviewed at baseline and at follow-up, and approved by the Regional Committee for Medical Research Ethics and by the Norwegian Data Inspectorate.

## Results

The prevalence of persons with SA in the six-year observation period was 19% (30/160). Table 1 show that patients younger than 30 years and those with only elementary school education were significantly more likely to have attempted suicide in the observation period. Ten per cent of abstainers were suicide attempters, compared with 22% of relapsers (*p *= 0.077).

**Table 1:** Frequency (%) of suicide attempters in the six-year observation period by socio-demographic variables and substance use variables measured at admission and at follow-up (N = 160).

Socio-demographic and substance use variables	Suicide attempters %
Age	
≥ 30 (n = 128)	16
<30 (n = 32)	31*
Gender	
Female (n = 47)	21
Male (n = 113)	18
Employed	
No (n = 96)	19
Yes (n = 49)	16
Married/cohabiting	
Yes (n = 56)	13
No (n = 89)	21
Elementary school only	
No (n = 85)	13
Yes (n = 75)	25*
Main substance of abuse	
Poly-substance-dependent (n = 74)	19
Alcohol dependent (n = 86)	19
Age of onset	
Substance use disorder	
≥ 18 years (n = 87)	16
<18 years (n = 73)	22
Use of substances at follow-up	
Abstainers (n = 48)	10
Relapsers (n = 112)	22

*p < 0.05

The mean number of lifetime Axis I disorders at index admission was 3.6 ± 2.6 (not in the table), and the frequency of SA in participants of each Axis 1 disorder is shown in Table 2. Table 2 also shows that patients with dysthymia were significantly more likely to attempt suicide in the forthcoming six years, whereas patients with Generalized Anxiety Disorder (GAD) were significantly less likely to attempt suicide. There was also a non-significant tendency towards a higher proportion of suicide attempters among patients with social anxiety disorder, bipolar disorder, somatization disorder and eating disorder. Only a small number of patients had bipolar disorder and eating disorders, so there is a risk of type II errors. When using current Axis I disorders (last 12 months) instead of lifetime Axis I disorders, dysthymia was still significantly associated with suicide attempts (28% vs. 14%, *p *= 0.025), as were all affective disorders (26% vs. 10%, *p *= 0.014). There was only a tendency (11% vs. 18%, *p *= 0.402) toward lower frequencies of suicide attempts in those with GAD, compared with patients without GAD. Twenty-four per cent of patients with SA at the index admission repeated the SA (20/84). There was a weak association between lifetime SA measured at index admission and new SA in the six-year observation period (24% vs 13%, *p *= 0.086).

**Table 2:** Frequency (%) of suicide attempters in the observation period by different lifetime Axis I disorders (N = 160).

Axis I disorders	Suicide attempters %
Any Axis I disorder	
No (n = 15)	13
Yes (n = 145)	19
Bipolar	
No (n = 145)	17
Yes (n = 7)	43
Major depression	
No (n = 85)	20
Yes (n = 72)	18
Dysthymia	
No (n = 98)	12
Yes (n = 57)	28*
Agoraphobia with and without panic attack	
No (n = 87)	20
Yes (n = 70)	19
Social anxiety disorder	
No (n = 80)	14
Yes (n = 74)	26
Generalized anxiety disorder	
No (n = 129)	22
Yes (n = 31)	7*
Simple phobias	
No (n = 86)	16
Yes (n = 72)	21
Obsessive-compulsive disorder	
No (n = 145)	19
Yes (n = 15)	20
Post-traumatic stress disorder	
No (n = 124)	21
Yes (n = 34)	12
Somatization	
No (n = 115)	16
Yes (n = 44)	27
Eating disorder	
No (n = 143)	18
Yes (n = 13)	39
Number of Axis I disorders	
0 (n = 15)	13
1–3(n = 69)	17
4+ (n = 76)	21
Lifetime suicide attempts at T1	
No (n = 76)	13
Yes (n = 84)	24

*p < 0.05

Persons who attempted suicide in the observation period had a significantly higher score on the HSCL-25 at T1 (mean = 2.31 ± 0.63 in SA patients vs. 2.02 ± 0.59 in non-SA patients, *p *= 0.022). The SA patients also had significantly higher scores on HSCL-25 at T2 (mean = 2.31 ± 0.55 in SA patients vs. 1.92 ± 0.63 in non-SA patients, *p *= 0.002). Patients who were treated in the mental health system during the observation period reported a SA significantly more often (30% vs 8%, *p *< 0.001).

The mean number of Axis II disorders was 2.7 ± 2.6 (not in the table). The frequency of SA in participants with each Axis II disorders is shown in Table 3. Four of the thirteen Axis II disorders were significantly associated with SA: paranoid personality disorder (PD); schizotypal PD; borderline PD; and passive-aggressive PD. The number of PDs was significantly associated with SA (OR = 1.24, *p *= 0.006). When using MCMI-II dimensionally, only paranoid PD was significantly associated with SA. Comorbidity of Axis I and II (patients who simultaneously satisfied the criteria of one or more Axis I and Axis II disorders, n = 100) was not significantly associated with SA in the observation period (23% vs 14%, *p *= 0.204). However, bivariately, the total number of Axis I and II disorders was significantly associated with SA in a logistic regression analysis (OR = 1.1, *p *= 0.013).

**Table 3:** Frequency (%) of suicide attempters in the observation period by different Axis II disorders (N = 149).

Axis II disorders	Suicide attempters %
Any Axis II disorder	
No (n = 41)	15
Yes (n = 108)	22
Paranoid	
No (n = 143)	18
Yes (n = 6)	67**
Schizoid	
No (n = 124)	19
Yes (n = 25)	28
Schizotypal	
No (n = 129)	17
Yes (n = 20)	40*
Antisocial	
No (n = 102)	17
Yes (n = 47)	28
Borderline	
No (n = 102)	15
Yes (n = 47)	32*
Histrionic	
No (n = 132)	20
Yes (n = 17)	24
Narcissistic	
No (n = 130)	21
Yes (n = 19)	16
Avoidant	
No (n = 92)	16
Yes (n = 57)	26
Dependent	
No (n = 122)	19
Yes (n = 27)	26
Compulsive	
No (n = 141)	21
Yes (n = 8)	13
Passive-aggressive	
No (n = 90)	13
Yes (n = 59)	31*
Self-defeating	
No (n = 110)	16
Yes (n = 39)	31
Aggressive-sadistic	
No (n = 115)	18
Yes (n = 34)	27
Number of Axis II disorders	
0 (n = 41)	15
1–3 (n = 54)	13
4+ (n = 54)	32*

*p < 0.05

**p < 0.01

To further investigate whether lifetime Axis I and II disorders had an independent impact on being a suicide attempter in the follow-up period, a logistic regression analysis was conducted. The dependent variable was being a suicide attempter in the follow-up period (1 = yes and 0 = no). Two blocks of independent variables were entered. In the first block, only T1 variables that were significantly associated with SA in the bivariate analyses in Tables 1, 2 and 3 (i.e., dysthymia (1 = yes; 0 = no), GAD (1 = yes; 0 = no), number of Axis II disorders (continuous 0–9). In addition possible confounding socio-demographic variables age (continuous), gender (1 = men; 0 = women) and level of education (1 = elementary school only; 0 = more than elementary school) were controlled for. To examine whether some of the effect of T1 variables occurred through use of substances at T2, the variables "relapser" (1) and " abstainer" (0) were entered as block two. In separate analyses, the variable measuring the total number of Axis II disorders was replaced with each of the different Axis II disorders that were significantly associated with SA in Table 3 (1 = yes; 0 = no).

Table 4 shows that, in the first block, lifetime dysthymia (OR = 2.8, *p *= 0.029) increased the risk of SA in the follow-up period, while lifetime GAD reduced the risk of SA in the same period (OR = 0.2, *p *= 0.045), when socio-demographic variables and number of PDs were controlled for. Dysthymia and GAD showed a moderate, positive correlation with each other (OR = 2.6, *p *= 0.022). Patients with GAD more often had compulsive PD (OR = 4.9, *p *= 0.033), and PDs from cluster C (OR = 4,3, *p *= 0.033), and less often had PDs from cluster B (OR = 0.23, *p *= 0.014). There was no interaction between GAD and the personality disorders associated with SA.

**Table 4:** Lifetime Axis I and II disorders as predictors of suicide attempters in the six-year observation period when controlling for age, sex, education and substance use at T2. Logistic regression: method enter, blockwise (N = 145).

	Block 1			Block 2		
Predictors	Sig	OR	95%CI	Sig	OR	95% CI

Dysthymia	0.029	2.8	(1.1–7.1)	0.040	2.7	(1.0–7.0)
Generalized anxiety disorder	0.045	0.2	(0.04–0.9)	0.033	0.2	(0.03–0.9)
Number of Axis II disorders	0.118	1.2	(0.9–1.4)	0.169	1.1	(0.9–1.3)
Age (continuous)	0.574	0.9	(0.9–1.0)	0.712	0.9	(0.9–1.0)
Sex (male = 1)	0.855	0.9	(0.3–2.5)	0.774	0.9	(0.3–2.6)
Only elementary school (1 = yes)	0.139	2.0	(0.8–5.2)	0.076	2.4	(0.9–6.3)
Substance use at T2 (relapser = 1)				0.063	3.1	(0.9–10.5)

Overall percentage = 81.4

Abbreviations: OR, odds ratio; CI, confidence interval

In a series of multivariate analyses, the HSCL-25 score at T1, number of Axis I disorders, and the total number of Axis I and II disorders were not independently associated with SA in the observation period. None of the specific Axis II disorders or number of Axis II disorders was independently associated with SA in the observation period. Entering use of substances at T2 in block two did not change any of the associations found in block one. Substance abuse at T2 was not significantly and independently associated with SA, when AUDIT and DUDIT was used either in a categorical way or in a dimensional way. Controlling for SA at index admission did not change any of the associations in the final model.

## Discussion

### Factors associated with being a suicide attempter in the observation period

Nearly 20% of our help-seeking SUD patients reported at least one suicide attempt during the six-year observation period. This is a high rate of SA compared with rates in the general population. A 13-year prospective study of a community sample showed an SA rate of 1.9% [48]. Our finding is comparable with two other five-year prospective studies with SUD patients. A study of drug addicts from Norway [12], and a study of a mixed sample of alcohol and drug-dependent patients from Sweden [49], found 27% and 21 % SA, respectively. Several non-Scandinavian samples have reported lower SA rates. In a five-year follow-up of well-integrated alcohol-dependent patients with fewer comorbid psychiatric disorders, the SA rate was 5% [17]. In short-term follow-up studies (1–3 years) the SA rate varies from 5% to 12% [18-20,23,50]. A possible reason for the higher SA rate in the Scandinavian studies is the higher prevalence of Axis I disorders in these non-selective samples [38], which possibly include more poly-substance-dependent subjects [25]. Our study supports the notion that treatment-seeking SUD patients are at high risk for further suicide attempts.

Socio-demographic risk factors for SA in SUD populations parallel those reported in the general population [19,24,35,51] with an over-representation of women, young people, single, divorced and unemployed participants. In the multivariate analyses, there was only a tendency towards an association between low education and SA. Petronis et al. [52] reported a tendency towards educational achievement being inversely associated with SA in a population-based study, whereas Darke et al. [3] found less education to be significant and independently associated with SA in a study of heroin-dependent patients.

In contrast to the lifetime occurrence of SA at the index admission [25], our prospective data show equal proportions of SA in the alcohol-dependent patients and poly-substance-dependent patients. None of the admission SUD variables were associated with SA in the forthcoming six years.

We found no significant differences in the frequency of SA between abstainers and relapsers. In the multivariate analysis, there was only a tendency towards an association between harmful use of substances at follow-up and SA in the observation period. Preuss et al. [17] found the number of alcohol dependence criteria met during the five-year observation period to be an independent predictor of SA among alcohol-dependent patients. Darke et al. [19] found poly-substance use at baseline and during follow-up to be higher in SA participants who were heroin dependent. However, a lack of association between SUD recovery and suicide attempts has been reported in both alcohol-dependent patients [53] and in drug-dependent patients [18,21,54]. It is important to remember that former clients who had not used substances in a harmful way during the year preceding follow-up had frequencies of SA in the six-year observation period that were two to three times higher than the lifetime prevalence in population studies. Thus, sober SUD patients may be a risk group concerning suicide attempts because they seem to be more strongly linked to other Axis I and II disorders than substance abuse. While not prospective in nature, data on the patients' use of substances during the year prior to follow-up are important to report. These analyses differ from the longitudinal design of this study, because the association between SA and use of substances in the last year is cross-sectional.

In this study, we used the lifetime version of Axis I disorders instead of current Axis I disorders because we wanted to compare the results with a previously published cross-sectional study from the admission sample, which also used the lifetime version of Axis I disorders. Another reason was that with the current version (last 12 months), we would lose statistical power due to the lower prevalence rate of Axis I disorders. Comparable with Driessen et al.'s [23] findings, the association between lifetime SA and lifetime Axis I disorders were stronger in the cross sectional (and retrospective) part of our study [25], where all the specific Axis I disorders except GAD were significantly associated with lifetime SA. A much shorter observation time (lifetime versus six years), and lack of statistical power in the prospective part, may be reasons for this discrepancy. Major depression, agoraphobia and eating disorders were each independently associated with lifetime SA at admission. In the prospective study, only dysthymia was significantly associated with an increased risk of SA. Together, the cross sectional and prospective parts of the study indicate that affective disorders, especially the chronic, long lasting dysthymic disorder, are stable predictors of SA in SUD clients, a finding supported by the research literature. The results from the analysis using current Axis I disorders underscore the importance of affective disorders as predictors of SA in prospective studies. Thus, affective disorders should be treated independently of substance abuse to reduce the risk of further suicide attempts.

GAD's protective function vis-à-vis SA was a finding that, as far as we know, has not been documented before. The positive correlation between cluster C PDs and GAD, and the negative correlation with cluster B PDs, may indicate that GAD is possibly related to avoidant behaviour and a higher impulse control and thereby indirectly related to a low rate of SA. This finding certainly needs replication and further exploration to determine the mechanism.

Only a few studies have assessed the influence of Axis II disorders on SA in SUD samples [12,13,23,32]. A main finding is that PDs in general, and borderline [3,12,32] and antisocial PD in particular, are associated with SA [6,7,13,23,26,55]. In the current study, the predictive value of personality disorders was limited. One reason for this might be the lack of statistical power. We need to be aware of the small number of patients who had some of the Axis II disorders, which will decrease the likelihood of detecting significant findings (type II error). The wide confidence intervals reported in Table 4 are consistent with this assertion.

However, the lack of a predictive effect of Axis II disorders has been shown in another five-year prospective study from Norway using MCMI [12]. A possible reason for the lack of predictive value may be that MCMI-II in help-seeking, distressed SUD patients' measures state more than traits. Another reason could be that MCMI was used in a categorical way [56]. However, in the current study, we also used MCMI in a dimensional way without any changes to the results.

In the multivariate analyses, the number of Axis I disorders, number of Axis II disorders, and the sum of all these disorders were not independently associated with SA in the observation period. This indicates that the associations between lifetime Axis I and II disorders and SA were weaker in the prospective study than in the cross-sectional study, which is in contrast with findings from cross-sectional population-based studies and most clinical studies. In a study of the prevalence of, and risk factors for, lifetime suicide attempts in the National Comorbidity Study, Kessler and colleagues [4] found the number of Axis I disorders to be a significant predictor of SA, over and above the effects of specific disorders. In addition, a study of comorbidity patterns in German adolescents and young adults who had attempted suicide showed that the comorbidity of the number of diagnoses, and especially comorbidity with multiple diagnoses (anxiety plus depressive disorders), was strongly associated with SA [27].

In clinical studies, there are contradicting results on the influence of specific Axis I disorders contra a general psychiatric severity measure (as number of disorders) and the association with suicide attempts. Cross-sectional studies of alcoholics, [6,7,26] found an association between the number of Axis I disorders and an increased risk of SA. However, in a 2 1/2-year prospective study of opiate addicts, Kosten and Rounsaville [20] found specific diagnoses (RDC diagnosis) to be better than a global measure (the ASI psychological scale) in predicting SA. It is difficult to compare this study with others because the number of disorders was not used as a variable. In cross-sectional studies with long observation times (lifetimes), a higher number of disorders might reflect a higher burden for the patient, resulting in a higher risk of attempting suicide. Another possibility is that multiple psychiatric disorders might interact with each other to produce a high risk of attempting suicide. In conclusion, in our study the lifetime individual disorder and current dysthymic disorder were better predictors of SA than the sum of all Axis I and II disorders. Thus, our study does not support the notion that it is the general level of psychopathology, and not affective disorders specifically, that matters.

Substance-dependent participants carry a higher risk for repeating SA than non-substance-dependent subjects [22,53,56,57]. In the present study, a SA measured at index admission was not significantly associated with a new SA in the observation period, as reported in many other follow-up studies in alcohol-dependent patients [17,23,32,53] and in drug-dependent samples [12,18,19]. This discrepancy may be caused by the fact that many samples consist of patients who have been hospitalized due to their SA. Thus, their SA have been serious. In contrast, we know nothing about the suicide method, the medical injury or the level of suicide intent in the SA patients. Alcohol-dependent participants with SA have been found to show less serious intent, and have more impulsive attempts, with more lethal methods than those without alcohol dependence [58], or those with major depression [59].

### Limitations

A strength of this study is the consecutive sample of treated SUD inpatients and outpatients that included both men and women, and alcohol-dependent and poly-substance-dependent participants. The use of structured interviews and reliable self-report instruments is also a strength, as is the prospective design with a six-year observation period.

However, there are some limitations. The results from this study rely on self-reported responses to a single yes/no question. Studies carried out in emergency rooms, general hospitals or in psychiatric settings tend to be defined with more precision [60]. Another shortcoming in the current study is the lack of information, in the follow-up period, about negative life events or setbacks in employment, physical health, social isolation, partnership difficulties, and financial difficulties factors that have been associated with SA in SUD patients [10,14,19,21,24,35]. There is also a lack of confirmatory information regarding the patients' use of substances and mental health variables. We had to rely on the patients' willingness to report the truth. However, many recent studies have supported the validity of self-reports of substance use [61].

Recall bias is an important problem, and over- and under-reporting of SA is a known phenomenon. In the current study, 13% (20/160) gave inconsistent responses about lifetime SA, which is less than has been reported in a prospective population study of adolescents, where one-third of all baseline suicide attempters did not report their attempt again at the four-year follow-up [62].

Representativeness is another problem, and in the present study, the follow-up sample constitutes only 23% of those patients who fulfilled the inclusion criteria in the baseline study (160/690), and 63% of surviving patients from the baseline sample (160/254). In the cross sectional study, there was an under-representation of young drug-dependent patients. In the follow-up study there were no differences between the follow-up sample (n = 160) and dropouts (n = 94) with regard to variables measured at baseline. With more young patients, the SA rates would have increased. However, in the multivariate analysis, age was not significantly associated with SA. Patients with SA in the observation period were more distressed than non-SA patients and we could not rule out the possibility that a higher proportion of SA/distressed patients refused to participate in the follow-up study.

## Conclusion

One in five participants made a SA in the six-year observation period, a relatively high proportion compared with other SUD samples. With the exception of affective disorders, the course of SA in the follow-up period, was less clearly associated with lifetime Axis I disorders than we previously found in a cross sectional study of the same sample [25]. Further, there were no associations between SUD variables measured at baseline, or at follow-up, and SA. Consequently, all SUD patients ought to be evaluated for suicidal risk, and particular efforts should be directed towards patients with comorbid affective disorders. The frequencies of SA in patients who had ceased their addictive behaviour was still very high compared with SA rates in the general population, a finding that also underlines the importance of treating more than just the addiction.

## Competing interests

The author(s) declare that they have no competing interests.

## Authors' contributions

KB undertook the conception and design of the study, the data collection, data analysis, and interpretation of the data, and drafted the manuscript. PV contributed to the conception and design of the study, to the drafting and revision of the manuscript, and has given final approval of the version to be published.
